# The expectations and realities of nutrigenomic testing in australia: A qualitative study

**DOI:** 10.1111/hex.13216

**Published:** 2021-02-26

**Authors:** Erin Tutty, Chriselle Hickerton, Bronwyn Terrill, Belinda McClaren, Rigan Tytherleigh, Elaine Stackpoole, Jaqueline Savard, Ainsley Newson, Anna Middleton, Amy Nisselle, Marie Cusack, Melissa Adamski, Clara Gaff, Sylvia Metcalfe

**Affiliations:** ^1^ Murdoch Children’s Research Institute Melbourne Victoria Australia; ^2^ Department of Paediatrics The University of Melbourne Melbourne Victoria Australia; ^3^ Australian Genomics Health Alliance Victoria Australia; ^4^ Kinghorn Centre for Clinical Genomics Garvan Institute of Medical Research Sydney NSW Australia; ^5^ St. Vincent’s Clinical School UNSW Sydney NSW Australia; ^6^ Sydney Health Ethics the University of Sydney Sydney NSW Australia; ^7^ Society and Ethics Research Connecting Science Wellcome Genome Campus Cambridge UK; ^8^ Faculty of Education University of Cambridge Cambridge UK; ^9^ Centre for Genetics Education NSW Health Sydney NSW Australia; ^10^ Department of Nutrition, Dietetics and Food Monash University Melbourne Victoria Australia; ^11^ The Walter and Eliza Hall Institute of Medical Research Melbourne Victoria Australia; ^12^ Present address: Genetic Services of Western Australia Subiaco WA Australia; ^13^ Present address: School of Medicine Faculty of Health Deakin University Geelong Victoria Australia

**Keywords:** complementary/alternative medicine, direct‐to‐consumer, *MTHFR*, nutrigenetics personal genomic testing, nutrigenomics, utritional genomics

## Abstract

**Background:**

Consumer genomic testing for nutrition and wellness, (nutritional genomics), is becoming increasingly popular. Concurrently, health‐care practitioners (HPs) working in private practice (including doctors interested in integrative medicine, private genetic counsellors, pharmacists, dieticians, naturopaths and nutritionists) are involved as test facilitators or interpreters.

**Objective:**

To explore Australian consumers’ and HPs’ experiences with nutrigenomic testing.

**Method:**

Semi‐structured in‐depth interviews were conducted using predominantly purposive sampling. The two data sets were analysed individually, then combined, using a constant comparative, thematic approach.

**Results:**

Overall, 45 interviews were conducted with consumers (n = 18) and HPs (n = 27). Many of the consumer interviewees experienced chronic ill‐health. Nutrigenomic testing was perceived as empowering and a source of hope for answers. While most made changes to their diet/supplements post‐test, self‐reported health improvements were small. A positive relationship with their HP appeared to minimize disappointment. HPs’ adoption and views of nutrigenomic testing varied. Those enthusiastic about testing saw the possibilities it could offer. However, many felt nutrigenomic testing was not the only ‘tool’ to utilize when offering health care.

**Discussion:**

This research highlights the important role HPs play in consumers’ experiences of nutrigenomics. The varied practice suggests relevant HPs require upskilling in this area to at least support their patients/clients, even if nutrigenomic testing is not part of their practice.

**Patient or public contribution:**

Advisory group included patient/public group representatives who informed study design; focus group participants gave feedback on the survey from which consumer interviewees were sourced. This informed the HP data set design. Interviewees from HP data set assisted with snowball sampling.

## 1 INTRODUCTION

In recent years, personal genomic testing or ‘consumer genomics’ has placed genomic information into the hands of consumers. These tests promise risk predictions in diverse areas including fitness, response to medications and traits such as premature balding. Also popular are tests for nutrition and wellness (nutrigenetic and nutrigenomic tests, hereafter referred to collectively as nutrigenomic tests), which offer diet and lifestyle recommendations that are personalized to genetic predisposition(s) to metabolic responses.^1^ Often advertised and sold online, these tests are marketed as being empowering and health transformative.^2^

Clinical validity (strength of the disease‐gene relationship) and clinical utility (impact on health outcome) are essential when evaluating any genomic test. Current Australian guidelines recommend against the use of nutrigenomic testing unless the test, and related dietary interventions, are sufficiently evidence‐based.^3^ Strong evidence does exist for certain gene–nutrient relationships, as is the case for diets low in phenylalanine prescribed to people with phenylketonuria, a genetic disorder of amino acid metabolism.^4^ However, for commercially available nutrigenomic tests, many of the selected gene–nutrient interactions, and the associated dietary advice, lack sufficient evidence to be considered clinically valid and useful.^5^, ^6^

Communicating the importance of clinical validity and utility becomes a challenge when genomic tests enter the consumer realm. An example of this is *MTHFR* gene testing.^1^, ^7^ The *MTHFR* (methylenetetrahydrofolate reductase) gene is responsible for the production of an enzyme involved in folate metabolism. Many online sources implicate two *MTHFR* single nucleotide polymorphisms (SNPs) in the risk of developing a myriad of health conditions and support the validity of testing and the utility of various supplements for prevention and treatment.^8^ However, a key international genetics organization^9^ and prominent consumer genomics company *23andMe* ^10^ recommend against *MTHFR* gene testing based on insufficient evidence. Nevertheless, clinical genetic services in Australian public hospitals have reported an increase in referrals for testing of *MTHFR* in recent years.^11^

Previously, genetic testing was reserved for diagnostic purposes in the context of inherited conditions and facilitated by genetic specialists or in primary care settings. Now, consumers can obtain nutrigenomic tests, including *MTHFR* gene tests, in a variety of other ways: some are available direct‐to‐consumer via online purchase and others via a ‘direct‐to‐practitioner’ model, where the process is facilitated by a health‐care practitioner (HP), who may have been trained by the relevant testing company.^12^ A recent content analysis of predominantly Australian websites revealed complementary/alternative medicine (CAM) practitioners, including naturopaths and nutritionists, are offering to facilitate testing or provide support regarding results interpretation.^2^ Other HPs in private practice known to provide these services include general practitioners (GPs) with an interest in CAM (‘integrative’ GPs) and those working in allied health, such as dieticians, pharmacists and private genetic counsellors (GCs).^2^ While GCs may not necessarily instigate this type of testing, in Australia there has been a substantial rise in consumer requests regarding consumer genomic testing, with 11% of genetic services reporting queries in 2011^13^ increasing to 66% in 2017.^14^ Additionally, in Australia, pharmacogenomic and nutrigenomic tests are sold by pharmacists over the counter in some community pharmacies.^15^

Published practice guidelines for GPs caution against the use of this testing,^16^ however, there is limited understanding of the practice of those in the fields of CAM and allied health.^17^ Further, little is known about consumer experiences of nutrigenomic tests, including those for *MTHFR*. The Genioz (Genomics: National Insights of Australians) study sought to explore consumer views and, where appropriate, experience with a variety of consumer genomic tests.^18^, ^19^, ^20^ Survey responses and follow‐up interviews revealed that some Australians are interested in and are pursuing nutrigenomic testing, often via a CAM practitioner.^19^, ^20^ These findings informed subsequent research to explore the landscape of HPs offering and/or interpreting consumer genomic tests. This additional work was part of the Workforce and Education Program of Australian Genomics Health Alliance (Australian Genomics), which focusses on the workforce implications of genomics in Australia.^21^ This paper presents the combined findings of these interviews with both consumers and HPs to explore their experiences with nutrigenomics in Australia.

## 2 METHODS

This study took a qualitative approach, comprising interview data from two data sets (Genioz to explore the experiences of the consumers and Australian Genomics to explore HPs’ experiences). Ethics approval was obtained for both studies (HREC 1 545 806.3, Genioz project; and HREC 1 646 785.9, Australian Genomics). The Genioz project was overseen by an advisory group including representatives of patient/public groups who advised researchers on study design and assisted with recruitment.

### 2.1 Participant recruitment

**FIGURE 1:**
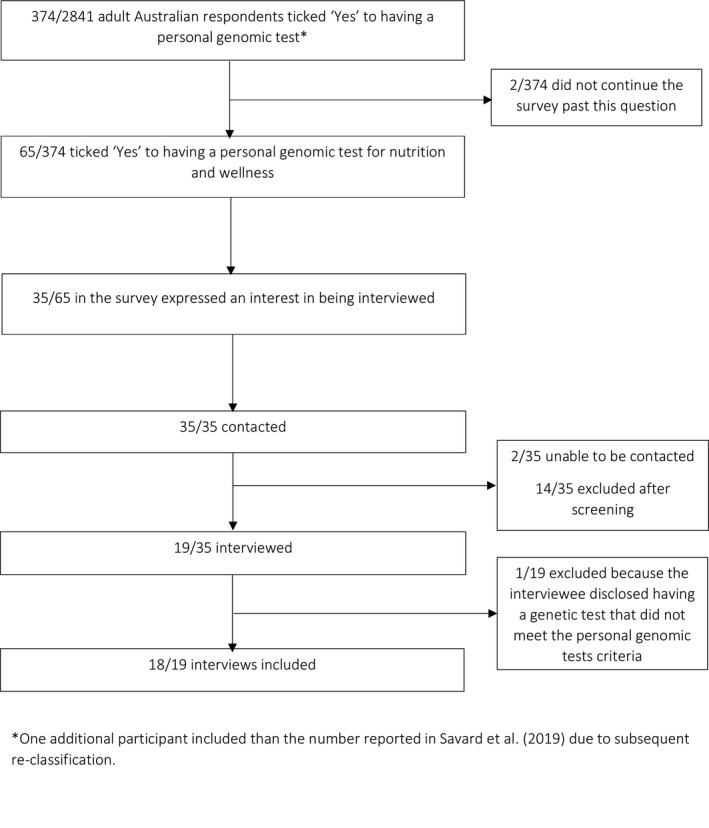
Consumer recruitment

Consumers were purposively sampled from Genioz online survey respondents (Figure 1).^20^ Survey respondents who indicated they would be prepared to be interviewed were sent an email. Given that survey respondents self‐selected the type of test they had pursued, screening calls took place to confirm eligibility. It became apparent that many potential interviewees had pursued single gene testing for *MTHFR* only. Therefore, as recruitment and data collection continued, additional individuals who had undergone testing for this gene only were excluded to ensure all aspects of the data set were well captured.^22^

Specific HPs working in private practice were identified as those who do or could offer nutrigenomic testing (informed by findings of the Genioz study).^20^ CAM (naturopathy, nutrition, integrative medicine) and allied health (dietetics, pharmacy and private genetic counselling) practitioners were recruited via four methods: purposive sampling after an online search to identify key individuals; random sampling through advertisements placed in practitioner‐specific professional society e‐newsletters; convenience sampling through professional research networks; and snowballing sampling through interviewee networks. Invitation letters were emailed, to which interested practitioners responded directly to the researcher.

### 2.2 Data collection

Consumer interviews explored the participants’ motivations for pursuing testing, their recollection of pre‐test information and receiving their results and any post‐test behaviour changes (Supplementary 1). Consumer interviews ranged in duration from 25‐75 minutes. Recruitment and data collection occurred iteratively between May 2016 and December 2017. HP interviews sought to understand the landscape of practice regarding consumer genomics, including nutrigenomics, awareness of this testing and its use in practice (Supplementary 2). HP interviews ranged in duration from 15‐123 minutes. Recruitment and data collection occurred between February and December 2019.

### 2.3 Data analysis

Audio recordings were transcribed verbatim and de‐identified, with pseudonyms assigned to consumers and ID numbers assigned to HPs. NVivo 11 and NVivo 12 (QSR International, Melbourne, VIC, Australia) were used for data management, for consumers and HPs respectively.

For each data set, transcripts were coded by ET (consumers) and CH (HPs) and analysed thematically, using an inductive, constant‐comparative approach.^23^ A sample of the transcripts was coded independently by at least one other researcher for each data set (CH and SM for consumers; AR and SM for HPs), from which a consensus was reached regarding the final themes. Coding frameworks were developed for each data set with their own set of themes identified. Themes were then compared by ET, CH and SM, imported into a new NVivo 12 file and coded further, resulting in common themes presented in this paper and agreed upon by the other authors (additional consumer themes were published in a Genioz Honours research thesis, and additional HP themes are to be reported in a separate manuscript). Coding was also conducted regarding HPs’ views of and engagement with nutrigenomic testing in their practice. This resulted in a detailed matrix of the landscape of practice for all HPs, which was then visually represented as four quadrants. Four of the researchers (CH, BT, ET and SM) reached consensus with the placement of HPs in each specific quadrant based on each HP’s views of, and level of engagement with, nutrigenomic testing described in their interview.

## 3 RESULTS

### 3.1 Participant characteristics

**FIGURE 2:**
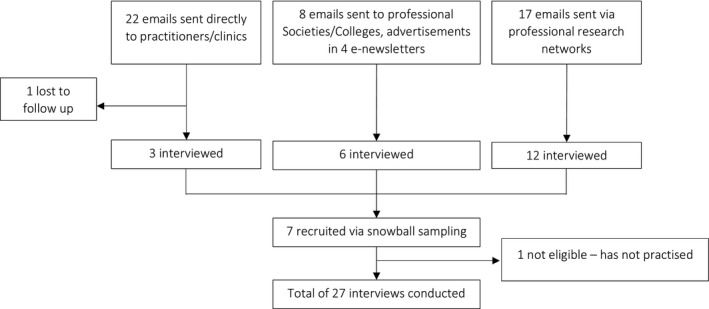
Health‐care practitioner recruitment

**FIGURE 3:**
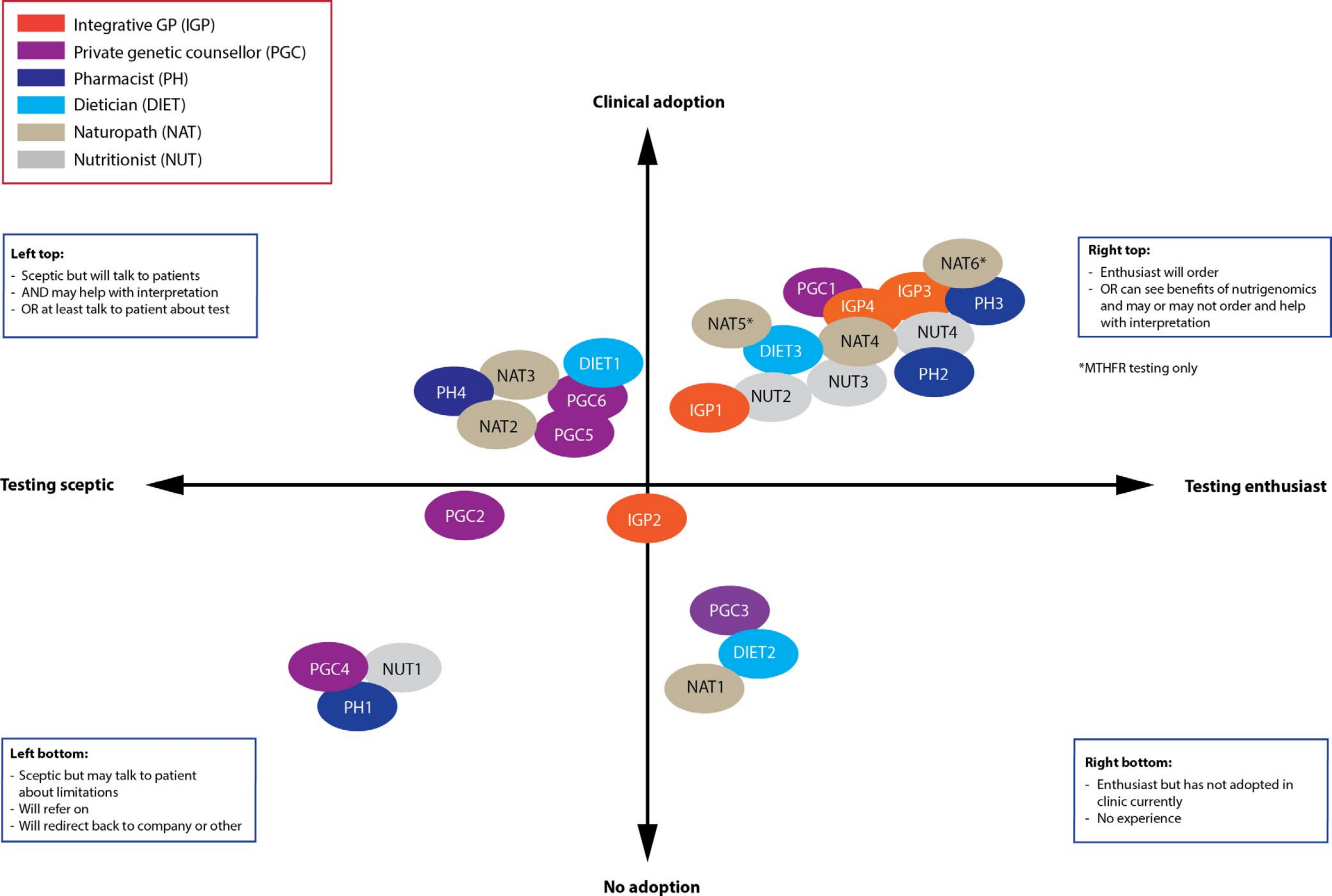
Visual representation of the variability between HPs in their practice of nutrigenomics. Clinical adoption: from testing and interpretation (top) to referring (in between) to no adoption where this testing does not fit into a practitioner's current practice (bottom). Each HP was coded as enthusiastic, sceptical or in between according to their views on testing. The same process was conducted for HP engagement with testing.

Eighteen consumers (Figure 1) and 28 HPs were interviewed (Figure 2). Participant characteristics are presented in Tables 1 and 2, respectively. In the consumer cohort, HPs first suggested nutrigenomic testing to eight consumers, while 10 consumers initiated their own testing and sought advice post‐test. While the HPs interviewed in this study were cautious about nutrigenomic testing, their views of, and practice around, nutrigenomic testing varied, as shown in Figure 3. Their views ranged from being sceptical (‘testing sceptic’: left quadrants) to enthusiastic (‘testing enthusiast’: right quadrants). There was also a mix of engagement with testing provision: whether they adopted testing in their practice, including ordering a test and/or interpreting test results (‘clinical adoption’: top quadrants), or reported little or no engagement (‘no adoption’: bottom quadrants). For example:

The DNA test, it actually zones in a little bit more … I send them off to the chemist to get them…it made me go, oh great, I’ve got science answering my question. (Nutritionist 4, right top quadrant)

I get a lot of email requests saying, ‘I heard you’re in this space and I want a genetic test to do X, Y and Z’ and I have to say, ‘that’s not something I can help you with’ and it’s also hard, you’ve got to be careful with your wording, saying, ‘I don’t recommend that’ without actually having seen them, but you can just tell from the email that a lot of it is not an evidence‐based test yet… if people already have results then I have seen them to be able to help them understand what it means to them in the context of their health goal and whether there’s any useable information in there or not. (Dietician 1, left top quadrant)

I don’t really feel like it really fits into my practice currently. That’s not to say it won’t in the future…if they [client] brought that in I would have no problem with that. I wouldn’t dismiss it as being invalid or not of any use but I would then look at the company that has carried out the testing and go from there… (Naturopath 1, right bottom quadrant)

My personal and professional opinion would probably be I don’t know why someone would want to do that [nutrigenomic testing] of their own accord… (Pharmacist 1, left bottom quadrant)

**TABLE 1:** Consumer characteristics

	ID	Pseudonym	Gender^a^	Ethnicity^a^	Age range (years)^a^	Highest education level^a^	SEIFA^b^ by postal area code^a^	Self‐reported health (SF‐36^c^)^a^	Time between testing and interview	Experience with nutrigenomic testing
Testing first suggested by a health‐care practitioner	2405	Christopher	Male	Australian & N European	50—59	College/ other tertiary institution	6	Poor	3 years	Naturopath recommended nutrigenomic test. Christopher also did his own research and ordered *23andMe* online for health additional information. He now takes a variety of supplements.
	2380	Angela	Female	Australian & S European	30—39	University	5	Poor	5 years, 4 years	Integrative GP^d^ suggested a nutrigenomic test. A naturopath suggested Angela order *23andMe* online for additional information. She is on a tailored diet.
	2374	Daniel	Male	Australian	50—59	College/ other tertiary institution	6	Fair	3 years	Naturopath suggested an *MTHFR* gene test. Daniel later ordered *23andMe* test online for health information after researching it. He sought advice from a second naturopath. He takes supplements and natural medicines.
	1900	Connor	Male	Australian	40—49	Secondary school	8	Very good	Unknown	Heard about nutrigenomic testing and asked a naturopath for advice. The naturopath recommended a test (she had done this test herself) and ordered it for Connor. He has made minor changes to diet.
	2365	Dylan	Male	Australian	40—49	University	6	Fair	3 years	Went to CAM^e^ practitioner who suggested nutrigenomic testing. Dylan is on a tailored diet and takes supplements.
	887	Bridget	Female	Australian	60—69	College/ other tertiary institution	2	Very good	3 years, 2 years	Saw a GP with an interest in nutrition who suggested doing a nutrigenomic test (for *MTHFR*) and to order *23andMe* online to capture genes not covered in the nutrigenomic test. Bridget also used a third‐party interpretation site^f^ and is working through results with her GP.
	2362	Natasha	Female	Australian	50—59	University	10	Poor	1 year	GP suggested that Natasha order *23andMe* online. Natasha did not take results back to her GP but used third‐party interpretation sites instead. She also went to a naturopath for advice. She now takes supplements.
	2390	Madeleine	Female	Australian & N European	70—79	University	4	Good	Unknown	GP ordered *MTHFR* gene test. Madeleine then sought advice from a naturopath. She is on a tailored diet and takes vitamin B12 supplements.
Testing was consumer initiated	2366	Lucy	Female	Australian	20—29	Currently studying at college/ other tertiary institution	10	Fair	Unknown	Bought *MTHFR* gene test online, then sought advice from a naturopath. Lucy is on a tailored diet. Naturopath also cut down her supplement intake.
	2396	Beth	Female	Australian	70—79	College/ other tertiary institution	10	Excellent	4 years, 3 years	Bought *MTHFR* gene test and *23andMe* online and then took results to naturopath. Beth is on a tailored diet and takes supplements.
	2370	Allison	Female	Australian	40—49	University	8	Poor	3 years	Used online programmes then sought advice from a naturopath. Through her own research Allison found and ordered *23and Me* test online. She used third‐party interpretation sites, then saw a second naturopath. She is on a tailored diet and takes supplements.
	2376	Grace	Female	Caucasian African	60—69	College/ other tertiary institution	10	Poor	Unknown	Through her own research Grace found and ordered *23andMe* online. She used a third‐party interpretation site and then took results to a naturopath. She has made dietary changes.
	555	Isaac	Male	Australian	20—29	University	10	Very good	1 year	Found out about *23andMe* in an article and ordered online. Then Isaac bought three additional tests (including one for ancestry). Then used third‐party interpretation site for further health information. No health‐care practitioner involved. No significant changes to diet.
	2008	Emilia	Female	Australian	50—59	University	4	Excellent	4 years	Received *23andMe* test as a gift. Emilia took her health results to GP (who didn't know what to do with results) and then also uploaded to third‐party interpretation site. She has made minor changes to diet.
	807	Hanna	Female	Australian & N, S & E European	60—69	University	6	Fair	4 years	Hanna uploaded her uploaded raw *23andMe* data to third‐party interpretation sites to obtain health information. She did not take results to health‐care practitioner. Later, she uploaded raw data to another third‐party site and gave results to naturopath. She takes supplements.
	2164	Pearl	Female	Australian	70—79	University	2	Good	Unknown	Asked GP to order *MTHFR* test. Planned to see naturopath.
	2378	Alexandra	Female	Australian	30—39	University	6	Fair	Unknown	First bought ancestry test online and then ordered an *MTHFR* gene test online. Alexandra took her results to her GP who was not interested, so she went to a nutritionist instead. She has changed her diet.
	2126	Samira	Female	Aboriginal/Torres Strait Islander & Maori/Pacific Islander	50—59	University	2	Fair	Unknown	Bought ancestry testing online first then had clinical genetic testing through GP. Samira also uploaded her raw data to third‐party interpretation site and took results to GP. She has made minor changes to diet.

^a^ Self‐reported data from survey.

^b^ SEIFA (IRSAD) – Socio‐Economic Indexes for Areas (Index of Relative Socio‐Economic Advantage and Disadvantage) – ranks areas in Australia by postal area code according to relative socio‐economic advantage and disadvantage (1 = most disadvantaged; 10 = most advantaged), based on Australian Bureau of Statistics 2011 census (most relevant to time of survey)^40^.

^c^ Self‐reported health based on SF‐36^41^: poor, fair, good, very good, excellent, unknown.

^d^ A general practitioner (GP) who incorporates complementary/alternative medicine into their practice.

^e^ Complementary/alternative medicine.

^f^ Third‐party online applications for interpreting raw genomic data obtained from personal genomic testing.

**TABLE 2:** Health‐care practitioner characteristics

Health‐care practitioner type	Range of years of practice	Have had a consumer test for self	Tertiary training in genetics	Training in nutrition
Naturopath n = 6	Recently graduated‐>20 years	Yes: n = 2 No: n = 3 Not stated: n = 1	No: n = 4 Yes: n = 2 (basics)	Yes: n = 6
Nutritionist n = 4	7‐16 years	Yes: n = 1 Not stated: n = 1 No: n = 2	No: n = 2 Yes: n = 2 (basics)	Yes: n = 4
Integrative general practitioner n = 4	13‐>30 years	Yes: n = 1 No: n = 3 (1 offered but declined)	No: n = 2 Yes: n = 2 (basics)	No: n = 2 Yes: n = 2 (1 taken courses with professional bodies)
Private genetic counsellor n = 6	7‐25 years	No: n = 5 Yes: n = 1	Yes: n = 6	Yes: n = 1 No: n = 5
Dietician n = 3	5‐13 years	Yes: n = 1 No: n = 1 Not stated: n = 1	No: n = 3	Yes: n = 3
Pharmacist n = 4	3‐15 years	No: n = 1 Not stated: n = 3	No: n = 1 Yes: n = 2 (basics) Not stated: n = 1	No: n = 4

### 3.2 Themes

Three key themes were developed from the two data sets. Illustrative quotes are included in‐text, with additional quotes in Table 3.

**TABLE 3:** Additional quotes

Theme	Quote #	Participant	Quote
Nutrigenomics: offering a source of hope?	1	Daniel	*We'd tried everything… I just wanted answers, I just wanted to feel better*
	2	Angela	*I was really interested in having something that was evidence based, and that would allow me to get to the root cause of the problem, so it wasn't just this merry‐go‐round of tablets and illness*
	3	Grace	*I went to about five doctors who poo‐pooed it and said, ‘No, it's a load of nonsense.’*
	4	Dietician 1	*…there's such individuality between people that I believe this space* [nutrigenomics]*, I feel that this space has helped start to answer those questions…*
	5	Integrative GP 3	*…..so many ways of doing genomic testing that they come back with all different SNPs and different numbers of the genes and just because there's the one* MTHFR *gene* [in testing] *there's all these other ones to consider*
The varying practice and expectations of nutrigenomics in health care	6	Angela	*So I had a really good* [integrative GP], *who suggested that I might have methylation issues and to do a DNA test to find out, and just sort of get information. So … I think she suggested* [testing company]
	7	Connor	*She* [naturopath] *showed me briefly, she went through the results of her own ‐ like she'd have her own done*… *I don't think she'd done a lot of them*…*so she'd been able to discuss a little bit about what had come back on hers. So, it was very, very general information just on the kind of things that might come back*
	8	Allison	*I got in contact with* [a naturopath] *told them I’ve got my genetic profile, and they then helped me convert it into readable data through the* [online program]…*I had to pay for the* [online program] *to have the conversion done, and then I got the SNP information. And then I had… probably 6 to 8 months of consultations through them to try and analyse and figure out how to help me…*
	9	Naturopath 3	*I have had patients who have come to me having already done genomic testing and that will be either they've done that on their own accord such as accessing* 23andMe *and then the conversion software to turn that into something a bit more readable*
	10	Private GC 4	*I mean it's so hard…. a lot of people call us and say ‘oh I’ve had this test can I talk to you about a result’ and our answer is no. So we're not providing counselling to people who have accessed testing from other places, I know there are companies that do do that and at this stage we do not, so we're not a sort of a dial up genetics service, like an interpretation* [service].
	11	Naturopath 4	*…I usually just get clients to order their own. It's much easier. I direct them which one to order if we need to but then they go away, go to the website, order it, pay for it themselves*
	12	Dietician 3	*It's another tool we have access to as practitioners*
	13	Naturopath 3	*It's still something I’m absolutely on the fence with this….. I don't really know that we're the right profession. We've just taken up the baton on it, we are a very motivated bunch but ten years ago when this sort of started becoming available there were certain people in our field who just ran with it and have made enormous businesses out of it, which is not I think, 100% ethical, but as a profession we generally do run with things pretty early*.
	14	Private GC 2	*…part of me wants to say it should only be genetics people that should be providing this but…those days are gone, genetics is everywhere, I think more and more of our roles as genetic counsellors, it's going to be picking up the pieces, we can't sit down and do an in‐depth interview with someone for pre testing and these people are having tests, and I think it's up to us to really help people when mistakes happen or unexpected things come up…*
Balancing clinical utility and personal utility	15	Pharmacist 4	*I find the results are a bit wishy‐washy. You just don't know what they're really telling you… There's a lot of ifs and buts. It doesn't really help. I think it could be misconstrued by patients of then being low in certain vitamins and minerals. I’m not so sold on it personally*
	16	Dietician 3	*…the range of testing out there is very broad and there will be companies that will be doing random combinations that are just out to get money. I think it's dangerous*
	17	Naturopath 6 (special interest in *MTHFR*)	*I started doing the* 23andMe *and then looking at all the different SNPs…I started to put the pieces of the puzzle together…In fertility, I found out I could really, amazingly, turn around the fertility and stop these women having miscarriages by changing the form and the dose of the folate*
	18	Private GC 2	*…public hospitals are run off their feet, they do not need someone sitting down and talking to them about* MTHFR *for half an hour, whereas if people were happy to pay, I thought as a genetic counsellor in the private sector I would be perfect for that. There wasn't a huge demand for that service, I felt I had to be careful, like I didn't want to tell people look I think that you've completely wasted your money, it was a complete waste of time, you have to be respectful, but in the same time I could say I think there is limited research into this particular thing and I think that part of my role was…to go through it* [the report] *and just go bit by bit what they meant and then I would usually summarise, write a patient summary letter or a client summary letter with the pertinent points and then I’d send a copy to their GP, so I think it was education, part of my role was educating people and to help the GPs in that*
	19	Private GC 3	*I used to work as a genetic counsellor in* [city]…, *so say for example a woman called up and she was just devastated because she's received an* MTHFR *result saying that she was at risk for everything under the sun… and so my colleague said to her ‘where did you get this information from, who ordered this test?’ and it was a naturopath. And so my colleague posed as a member of the public and called the naturopath and asked her questions about* MTHFR *testing and would it help her to understand why she had had a miscarriage and why this and why that and the naturopath was like ‘yeah absolutely, you should come in and have your* MTHFR *testing done’. So with our science hats on, as a genetic counsellor we're just like but we know there's no good evidence around this and it's actually not a useful test at all and there's some publications on this*
	20	Dylan	*To finally have some answers as to what's going on, you know, like, it was the best thing I’ve ever done. Because I was able to start putting the pieces of my life together*
	21	Lucy	*It was amazing. It was so, um, I even get emotional talking about it now, because um, it was life changing, in the sense that you finally get a diagnosis, or a cause, you know? You find sort of a root cause to your life struggles, if you will, and that's a massive thing, you know?*

#### 3.2.1 Theme 1 – Nutrigenomics: offering a source of hope?

Thirteen of the consumer participants had a long history of health problems and were yet to find any answers or solutions to provide a diagnosis or to guide future treatment and management of their condition, as shown in Table 3: Quote 1 and also described below:

And I’ve seen so many different doctors and I’ve done the standard tests, you know, just the standard blood tests a million times, only to get the same sort of response from my doctors that I was all good, it was all fine. (Lucy)

Some consumers were unsure what to expect from nutrigenomic testing and described ‘*taking a leap of faith’* that testing would provide not only answers but tangible solutions to their health concerns (Table 3: Quote 2).

Several consumers first came across nutrigenomic testing while searching online and explained that they did not do much research into the nutrigenomic testing company, or the specific test, before undertaking testing. Allison said: '*I didn't really look that much into* [nutrigenomic testing]. *I just wanted my profile done so I could find out how to treat* MTHFR.'

Despite their own eagerness to pursue nutrigenomic testing, several consumers were discouraged from testing by their conventional HPs (Table 3: Quote 3) and reported that CAM practitioners appeared more willing to use nutrigenomic testing. The HP interviewees had varied views on what nutrigenomics could offer their patients/clients. Some expressed similar excitement as the consumers regarding the possibilities of this testing (Table 3: Quote 4). Some described instances in which they believed testing was useful:

I had a guy who was overweight…we got talking about this test and so he did the test and he came back and we did his results and one of his results was…to do with letting the brain know that you’ve eaten enough food…I think it was helpful for him. (Pharmacist 2)

Whereas other HPs spoke of the limitations of testing (Table 3: Quote 5) and the importance of understanding the patient's broader health circumstances:

I think that there is a lot of interesting stuff you can glean from a genetic test as long as you are really aware of the limitations of that test and you know how to put it in the context of the patient in front of you…unless you actually understand a whole heap about the patient you can’t do anything really useful with that… (Private GC1)

Health‐care practitioners also acknowledged their clients’ quest for answers, noting that nutrigenomic testing was often the last option to be explored:

They’re asking you to fix [them]. The guy that brought that [DNA testing] he’s got chronic fatigue symptoms, heavy metal poisoning and… was basically saying, ‘is there anything in there that’s stopping me getting better?’ Which he has done every test under the sun, the genetic test was the last one. (Nutritionist 3)

While consumers described nutrigenomic testing as a last resort and a final hope for answers, many wished they could have accessed testing much earlier to reduce emotional impact. They also hoped to avoid the trial‐and‐error they reported experiencing and which they felt was associated with conventional medical practice and standard CAM:

I wish I had known about this stuff a bit sooner because I have been absolutely put through the ringer by a lot of medical people. Even stuff to the point where it’s like, ‘Is this all in your mind?’ (Samira)

#### 3.2.2 Theme 2 – The varying practice and expectations of nutrigenomics in health care

Several consumers reported previous negative experiences with GPs and specialists during their health‐care journey, which influenced their perception of nutrigenomics. Many consumers actively sought out an HP who could facilitate testing or help interpret results. Beth explained:

The professor was discouraging me from [nutrigenomic testing]… I said, ‘I just want to do it because it makes sense.’ So that’s why I switched from him [to a naturopath]. (Beth)

These consumers saw an interest in nutrigenomics as a sign an HP may provide them with the care/support they desired. Other consumers explained that their CAM practitioner recommended nutrigenomic testing, including *MTHFR* gene testing, and they commented that they were happy to have testing with the specific company suggested by the HP (Table 3: Quote 6). Several consumers explained that their HP had had testing themselves, which increased their confidence in both the test and information they received (Table 3: Quote 7). Consumers reported limited pre‐test counselling, however and justified this by stating they had an existing relationship and trust in their health‐care practitioner:

I don’t know that he went into full‐on detail because…I’ve been seeing him, I’ve known him for a few years so he sort of, he didn’t have to explain it ‐ you know what I mean? …I have a good relationship with him so I trust his judgement on that. (Natasha)

Angela and Bridget reported that, following the results of their first nutrigenomic testing, they were advised by their HP to purchase additional tests, including for ancestry. This was, as Angela explained, to gain ‘*more information*’. Several consumers reported that they were advised to download their raw genomic data from ancestry genomic testing and use it to obtain health information from online third‐party interpretation programmes (Table 3: Quote 8). A few HPs had also mentioned helping consumers with third‐party interpretation reports (Table 3: Quote 9).

A few HPs, particularly, private GCs, felt testing did not fit within their practice and would advise clients to go elsewhere (Table 3: Quote 10). However, many had adopted nutrigenomics in their practice in some way: actively ordering testing or encouraging clients to order nutrigenomic testing online and bring the results back for interpretation (Table 3: Quote 11). Not all HPs felt they were equipped to talk to their patients/clients about nutrigenomic tests:

I’ll tell them to go back to the person who ordered the test because I don’t understand this, that my knowledge is limited and it wasn’t me who initiated the test and then to go back to the service who organised this because they will have a better idea. (Integrative GP 1)

Nevertheless, many HPs in this cohort were willing to have a conversation with their patients/clients and some felt it important not to dismiss the testing outright (Table 3: Quote 12), while a few practitioners questioned whether nutrigenomic testing has a place in their discipline (Table 3: Quote 13). Additionally, private GCs agreed nutrigenomic testing does not have a place in public genetic clinics:

I think Genetics [the profession] has been a bit lax in having a voice in this field…I do see GCs dismiss it too quickly and I think that they are, in dismissing the client, leave that client open to alternative therapists that are much less educated. (Private GC 1)

Some HPs have found this testing useful and explained that initially they were actively ordering and encouraging testing. Yet, as time has passed, they have found they now only turn to it for specific circumstances:

I'm using genetic testing less and less. If you’d asked me a couple of years ago, I would’ve been like, ‘Everyone gets a *23andMe*’…Now I only do it if we’re really looking for something in particular…I found that it wasn’t necessarily changing my course of action. It was justifying the course of action, but it wasn’t changing the course of action. (Naturopath 4)

Despite an awareness that nutrigenomic testing may offer hope to their clients, in practice HPs in this cohort said they would not always offer nutrigenomic testing in the first instance. HPs described nutrigenomic testing as one tool of many that they can utilize and not necessarily the first, or only, tool they would use:

There are certain practitioners in Australia, naturopathic practitioners, where their business revolves around genetics. In fact…I saw someone yesterday who had previously been seeing one of those practitioners, so they had all that information but for them that had gone a bit too far, it was just all about genetics and for them that was a bit of a negative now…It provides a few pieces of a bigger puzzle, it doesn’t give the answers. (Naturopath 3)

Beyond ordering testing and/or results interpretation, HPs also spoke of their role in managing patient/client expectations. They explained that without understanding the limitations of nutrigenomic testing, consumers may expect to receive all the answers they are looking for:

Another major factor is you’ve got to also think about the patients’ expectations, what is the question the patient is wanting answered… patients will say ‘I want to know what food I can and can’t eat’ and it doesn’t matter how accurate a genetic test is sometimes in regards to the nutrigenetics variation, they’re never going to answer that question. (Dietician 1)

A few HPs felt they were ‘picking up the pieces’ after consumers had had testing elsewhere (Table 3: Quote 14) and then came to them for help:

I think if the person themselves initiated because they’ve got some rationale in their own mind, and wanted to discuss it with me, I’d be happy to discuss the pros and cons with them…I’m finding myself largely in the position of the person who kind of like helping them to pick up the pieces of what that means in their life. (Integrative GP 2)

#### 3.2.3 Theme 3 – Balancing clinical and personal utility

Many HPs described the tension between supporting patients and questioning the clinical validity and utility of the testing and the trustworthiness of the testing companies (Table 3: Quotes 15‐16). This could place some HPs in an ethically challenging situation:

I actually feel a little bit apprehensive about it because I don’t know that I can see the validity within my own practice, or a little bit about the ethics does concern me too, I don’t really feel like it really fits into my practice currently. (Naturopath 1)

*MTHFR* gene testing posed the most challenges for the HP interviewees. While some were very confident in the validity and utility of *MTHFR* testing (Table 3: Quote 17), others were wary (Table 3: Quotes 18‐19). Several HPs spoke of balancing their clients’ enthusiasm with *MTHFR* testing with an acknowledgement of its limitations and spoke of using other methods of investigation instead:

If someone comes to you and they’ve got a problem with – they’ve got anxiety or they’ve got this and that …’I think I want an *MTHFR*’ … you just go, ‘All right. So, you’ve got *MTHFR* but what does that mean? So, what that means is, for me, I would then run a check on their histamine and homocysteine and look for markers of methylation to tell me which B vitamins, which folate, which B12, et cetera to give the person. (Naturopath 4)

Despite these varying views of HPs on the usefulness of nutrigenomics, most of the consumer interviewees relayed positive experiences with testing and *MTHFR* testing was reported as providing the answer for which many participants had been searching (Table 3: Quote 20). Receiving nutrigenomic test results was not only perceived to be empowering but consumers believed the results confirmed that their illness was not ‘*psychosomatic’*, despite what they had been told by their conventional HPs. Lucy said: '*To finally have someone acknowledge that and say, you know, ‘You're not crazy, there is actually something going on’… It's hard to put words to it, how big of a meaning that had for me*.'

As a result of their testing, some consumers reported making minor changes to their diets and others changed their supplement regimens. However, despite the perception that nutrigenomic testing gave them answers (Table 3: Quote 21), self‐reported improvements to health were limited for most. Some used the term ‘*trial and error’* to describe the process of finding the right combination and doses of supplements to take. Allison explained: '*It's been a very hard process… all the trial and error, and the wasted money and the wasted time…'*

Others found the information provided with the nutrigenomic testing results relatively unhelpful:

So…it suggested be careful with caffeine and alcohol and stuff …, what does that mean? Does that mean I shouldn’t, be drinking, full stop…Is that something that would just show up on everyone’s report, because good health advice is don’t drink too much…It’s hard to interpret …from a reasonable lifestyle perspective. (Connor)

Nevertheless, all consumers remained positive about their decision to pursue nutrigenomic testing and valued the fact that they had found an HP who was not only willing to work with their nutrigenomic result but also validated their health concerns. Thus, it appears that nutrigenomic testing had personal utility even in the absence of actionable results.

## 4 DISCUSSION

Nutrigenomic testing has grown in popularity in Australia, with interest from consumers and HPs. This may in part be due to the positively framed marketing that has circulated online.^24^ It appears that these online marketing strategies, portraying nutrigenomic testing as ‘health transformative’^2^ particularly resonate with people who are chronically unwell and those with an interest in CAM.

Most consumers in our study reported their health as being ‘poor’ or ‘fair’ and described themselves as being chronically unwell. Unlike early adopters of consumer genomic testing, who were primarily motivated by curiosity and an interest in the science,^25^ the chronically unwell consumers in this study saw nutrigenomic testing as a ‘means to an end’, having tried everything else and finding no answers to their health concerns. This is consistent with a previous characterization of CAM users.^26^ While nutrigenomic testing was not as health‐transformative as the consumer participants had hoped, they remained positive about their decision to pursue testing. The majority of consumers reported feeling empowered and validated after receiving their nutrigenomic testing results. This was particularly true for those who discovered they had a particular *MTHFR* SNP that they felt could explain their health concerns.

Early qualitative research exploring patient and doctor experiences of diagnosing *MTHFR* polymorphisms and providing treatments noted the vast array of self‐reported, unvalidated information online.^27^ In one study from the USA, patients reported the frustration of seeing many doctors throughout their lives and receiving no relief to their chronic symptoms and the majority described receiving their *MTHFR* test results as validating, empowering and life‐changing.^27^ Likewise, some of the chronically unwell consumers in our study perceived *MTHFR* SNPs to be the diagnosis for which they were searching. After receiving feedback from ‘mainstream’ HPs that nothing could be done for them, when participants received nutrigenomic testing results, they perceived it as providing tangible ‘solutions’ to their health concerns. Many reported little health improvements that could be explained as a direct result of nutrigenomic testing interventions, however, and the recommendations provided were often not the easy solutions that they had expected. Further, when considering the price of the test (ranging from AU$99 to more than AUD$500), the out‐of‐pocket fee for a consultation with a CAM provider or private GC, plus the costs of potential supplements, nutrigenomic testing can become a costly endeavour and may not be accessible to consumers with lower socio‐economic status.

Despite feeling disappointed, the chronically unwell consumers in this study maintained a positive attitude towards nutrigenomic testing. They reported benefit from the knowledge they gleaned from testing. There are varied published views regarding clinical and personal utility of genomic testing. Bunnik, Janssens and Schermer ^28^ state personal utility does not exist when there is no evidence of clinical utility. Conversely, Kohler, Turbitt and Biesecker ^29^ conducted a systematic review of ‘personal utility’ of genomic information (generally), finding that the information in itself is perceived as valuable, and has personal usefulness, to consumers. Other beneficial outcomes included enhanced self‐knowledge, feelings of control over the situation and the future, improved coping and well‐being and the fulfilment of curiosity about what the results may reveal.^29^ Thus, while nutrigenomic testing still led to ‘trial and error’ treatments in our cohort, results can provide consumers with a perception of knowledge and a sense of relief, hope and validation. However, the general population may not derive the same perceived benefits from nutrigenomic testing and may instead be at risk of disappointment.

The trusting relationship the consumers had with their HP who was involved with the nutrigenomic test (or test results) also mitigated disappointment with the test outcomes. Much of the positive experience of nutrigenomic testing therefore came from finding an HP who was willing to listen and did not immediately discount their chronic symptoms. This has been similarly described in the area of homeopathy.^30^ Given this, it is possible that the positive response to nutrigenomic testing relates less to nutritional genomic science and more to psychosocial factors including receiving support, empathy and validation from their HP. Having an HP who supports nutrigenomic testing, and has even had testing themselves, can add a sense of legitimacy to the test which may influence clients accordingly. HP‐facilitated testing is also one way of mitigating the possibility that consumers will misinterpret their test results. However, the range of disciplines and the varied nature of practice between HPs could prove problematic for consumers in choosing an HP. Additionally, concerns remain that HPs may be inadequately trained to facilitate informed decision making.^31^, ^32^ Given that some nutrigenomic testing companies train the HPs in‐house,^19^ this represents a potential conflict of interest and could translate to consumers receiving biased information and advice based on poor evidence.

A key tenet of genetic counselling, now a regulated profession in Australasia,^33^ is facilitating clients’ long‐term adaptation to genomic information.^34^ Genetic counsellors have traditionally been employed in hospitals within a public health system, counselling patients for tests ordered via an accredited laboratory. Scepticism regarding validity and utility of nutrigenomic testing, accompanied by GCs’ perception that commercial consumer genomic tests may not belong in the public health genetic clinic, is a valid concern. However, more recently, GCs are working in private practice^33^ and/or in collaboration with a variety of HPs outside of traditional genetic clinics. Private GCs are well placed to facilitate adaptation to nutrigenomic test results and provide the information and support that the consumers in our study desired.

Most GPs and allied health practitioners have limited knowledge of nutrigenomics and many also question the validity of testing.^35^, ^36^ Thus, consumers, as our cohort illustrated, have been turning to CAM, including integrative medicine, practitioners. It can be difficult for HPs to make sense of nutrigenomic results and indeed consumer genomic testing in general for patients.^37^, ^38^, ^39^ This research revealed discrepancies between consumers’ expectations of when to have a nutrigenomic test and when an HP might deem it appropriate. HPs also spoke of the need to mediate patient/client expectations and discuss the limitations of testing. When consumers are advertised to directly, limitations of testing are not generally highlighted.^2^

Many of the consumer interviewees experienced chronic illness and felt that a nutrigenomic test from the outset would have been preferable to their protracted and frustrating health‐care experience to date. However, it is also of note that despite many publications in just under the last two decades, there is still concern in the nutrigenomics field that the gap between experiments and evidence for health practice has not yet been achieved.^39^ More evidence‐based resources, summaries and recommendations for HPs to use have been advocated.^39^ Additionally, depending on their profession, HPs have many different tools to call upon when faced with supporting a client/patient. Some are unique to each discipline; however, most of the HPs in this study felt that nutrigenomic testing was the final tool to call upon.

Despite its contested evidence base, nutrigenomic testing is available to the public. There are clearly gaps between the expectations of consumers and the realities of what tools HPs may utilize to help find the underlying cause of a health issue. While the HPs in this cohort spoke of limitations and some discouraged testing, consumer data demonstrate that individuals may instead seek out a new HP who would facilitate testing. HPs’ support of and experience with nutrigenomic testing may also influence clients’ willingness to undergo testing.

### 4.1 Limitations

The majority of eligible consumers responded to the invitation for recruitment, however, given the nature of this process, it is likely not all types of experiences of personal genomics were captured in this cohort. Most consumer participants were relieved to discover an *MTHFR* polymorphism, but others who had a more negative experience may not have responded to the survey. Access to nutrigenomic testing may also vary based on ethnicity and socio‐economic status. Consumers in our study identified as primarily Australian (presumably Caucasian – noting that they could select more than one ethnic ancestry in the survey), highly educated, and resided in areas of middle to high socio‐economic advantage. While this is consistent with previous characterizations of CAM users,^26^ the views of individuals who are interested in this testing, but unable to afford it or for whom it may not be suitable due to poor ethnic coverage of reference data in some tests, may not have been captured.

While a broad number of different types of HPs were interviewed, few who currently actively order and promote nutrigenomic testing in their daily practice responded to an invitation to participate. Additionally, as each data set was collected at two different time points it may not reflect the landscape of HPs’ practice at the time of consumer interviews as highlighted by Naturopath 4 in Theme 2. Nevertheless, the findings provide a unique insight into both consumer and HP experiences of nutrigenomic testing.

## 5 CONCLUSION

This study provides insights into the motivations and experiences of consumers undertaking nutrigenomic testing and a snapshot of the landscape of practice and attitudes of a range of HPs in Australia. In particular, consumers with chronic ill‐health are motivated to have testing and make dietary/supplement changes post‐test. More research is needed to determine the extent to which these changes persist long‐term. Further, this research highlights the important role that HPs facilitating nutrigenomic testing play in the experience of test‐takers, while also emphasising the variability of practice and enthusiasm of testing across HPs and within each discipline. It is recommended that HPs who are likely to be approached by their patients/clients about nutrigenomics should increase their awareness of this testing to enable conversations and facilitate informed decision making, even if it is not part of their practice. Future research could explore the training currently available to HPs and any support or education they may require.

Currently, Australian guidelines and key international genetics organizations caution against the broad use of nutrigenomic tests. Nevertheless, our market society, paired with a strong rhetoric of consumer empowerment, has meant that individuals are accessing this type of testing. Further, the consumer participants in this study reported positive experiences with nutrigenomic testing despite the scientific community cautioning its use. Ultimately, it is imperative that appropriate and balanced information and support is available to those interested in nutrigenomic testing to ensure informed decisions are made. How such resources and supports are made available, however, is a greater challenge.

## 6 CONFLICT OF INTEREST STATEMENT

MA was a co‐supervisor of ET and involved in the research about the consumer experience, and was subsequently a participant in the health‐care practitioner data set. The remaining authors declare no conflict of interest.

## AUTHOR CONTRIBUTIONS

ET contributed to the conception, design, acquisition of data, analysis and interpretation of the consumer data. ET was also involved in interpretation of health‐care practitioner data compared with the consumer data and drafting this manuscript. ET approves of the version to be published. CH contributed to the design, acquisition of data, analysis and interpretation of both the consumer and health‐care practitioner data. CH co‐supervised ET as part of her Honours thesis and ES as part of her Master of Genetic Counselling thesis. CH was also involved in the drafting of this manuscript and approves of the version to be published. BT contributed to the conception and design of both the consumer and health‐care practitioner studies and was involved in discussions of interpretation of data. BT also contributed to, and critically revised this manuscript and approves of the version to be published. BM contributed to the conception and design of the health‐care practitioner study and was involved in discussions of interpretation of data. BM also critically revised this manuscript and approves of the version to be published. RT contributed to the conception, design, acquisition of data and early analysis of the consumer data. RT also critically revised this manuscript and approves of the version to be published. ES contributed to the conception, design, and acquisition of data and early analysis of the consumer data. CH and SM co‐supervised ES as part of her Master of Genetic Counselling thesis. ES also critically revised this manuscript and approves of the version to be published. JS contributed to the conception, design, and early analysis of the consumer data. JS also contributed to and critically revised this manuscript and approves of the version to be published. ANe contributed to the conception and design of the consumer study and was involved in discussion about interpretation of the data. ANe also contributed to and critically revised this manuscript and approves of the version to be published. AM contributed to the conception and design of the consumer study and was involved in discussion about interpretation of the data. AM also contributed to and critically revised this manuscript and approves of the version to be published. ANi contributed to the design of the health‐care practitioner study and was involved in discussions of interpretation of data. ANi also critically revised this manuscript and approves of the version to be published. MC contributed to the design of the health‐care practitioner study and was involved in discussions of interpretation of data. MC also critically revised this manuscript and approves of the version to be published. MA contributed to the conception and design of the consumer study and was involved in discussion about analysis and interpretation of the data. MA co‐supervised ET as part of her Honours thesis. MA also contributed to and critically revised this manuscript and approves of the version to be published. CG contributed to the conception and design of both the consumer and health‐care practitioner studies and was involved in discussions of interpretation of data. CG also contributed to and critically revised this manuscript and approves of the version to be published. SM contributed to the conception and design of both the consumer and health‐care practitioner studies and was involved in analysis and discussions of interpretation of data. SM co‐supervised ET as part of her Honours thesis and ES as part of her Master of Genetic Counselling thesis. SM also contributed to and critically revised this manuscript and approves of the version to be published.

## 7 ETHICS APPROVAL AND CONSENT TO PARTICIPATE

The study was approved by The University of Melbourne (HREC 1545806.3 and HREC 1646785.9), and informed consent was obtained from all participants. The study was performed in accordance with the Declaration of Helsinki.

## Supporting information

Supplementary S1

Supplementary S2

## Data Availability

The data that support the findings of this study are available on reasonable request from the corresponding author. The data are not publicly available due to privacy/ethical restrictions.
