# Supplementary material for: The expectations and realities of nutrigenomic testing in australia: A qualitative study
Source: Health Expect. 2021 Feb 26;24(2):670–86. doi: 10.1111/hex.13216 (PMC8077070; doi:10.1111/hex.13216)
Supplement: Supplementary file 1 — Supplementary S1 [file HEX-24-670-s001.docx]

**Supplementary 1: Interview guide domains for consumers^1^**

| **Domain** | **Questions** |
| --- | --- |
| Motivations to have nutrigenomic testing | How did you hear about it? |
|  | What stood out to you about it? |
| Pre-test | What did your doctor/naturopath/other tell you about the test? |
|  | Did you do any research online? |
|  | Did you have any concerns |
|  | What were your expectations of the testing process? |
|  | What were your expectations of the results? |
| Receiving results | What was it like to receive your results? |
|  | Did you feel you understood the information? |
|  | Were your expectations met? Why/Why not? |
| Post-test | Have you made any changes to your diet/lifestyle? What, how? |
|  | Have you shared your results with anyone? Online? |
|  | Would you recommend the program others? |
| Reflections | How do you feel about the testing experience now? |
|  | What does wellness mean to you? |

^1^ The Genoiz interview guide was developed collaboratively by members of the authorship team and went through iterative versions. Additional questions related to nutrigenomic testing were added after team discussion and were further refined as data collection progressed. This guide was also subject to technical review as part of the ethical review process.
