# Supplementary material for: The expectations and realities of nutrigenomic testing in australia: A qualitative study
Source: Health Expect. 2021 Feb 26;24(2):670–86. doi: 10.1111/hex.13216 (PMC8077070; doi:10.1111/hex.13216)
Supplement: Supplementary file 2 — Supplementary S2 [file HEX-24-670-s002.docx]

**Supplementary 2: Interview guide domains for health practitioners^1^**

| **Domain** | **Questions** |
| --- | --- |
| Introduction | Background and current role |
|  | Experience with genetic/genomic testing |
| Description of past/current educational activities^2^ | What sort of educational activities have you attended about genetics or online DNA tests relevant for your practice? |
|  | Why did you attend the program? |
|  | Who delivered the program? |
|  | When was it held? |
|  | Where was it held? |
|  | What was the delivery mode? |
|  | Was it formally assessed/accredited? |
| Evaluation^2^ | Did the program meet your needs? |
|  | Did the delivery mode work for you? |
|  | Would you recommend the program to others? |
| Views on current and future practice | Can you tell me what you think about genetic/online DNA testing in terms of the patients you see in your practice? |
|  | What is your organisations (where you work or the profession) stance on personal genomic testing (online DNA testing)? |

^1^ The original interview guide was developed collaboratively by members of the Australian Genomics Workforce and Education Program authorship team. It was adapted for questions related to consumer genomic testing (including nutrigenomics). This guide was also subject to technical review as part of the ethical review process. This guide also underwent refinement as data collection progressed.

^2^ The findings for these questions are to be reported in a separate manuscript.
